# Level and factors associated with birth preparedness and complication readiness among semi-pastoral pregnant women in southern Ethiopia, 2016

**DOI:** 10.1186/s13104-018-3539-7

**Published:** 2018-07-04

**Authors:** Amanuel Iyasu, Mulatu Ayana Hordofa, Haymanot Zeleke, Cheru Tesema Leshargie

**Affiliations:** 1 Maternal and Child Health Program, Bule Hora District Health Office, Borena Zone, Ethiopia; 2 Department of Public Health, Health Science College, Debre Markos University, P.o.box: 269, Debre Markos, Amhara region Ethiopia; 3 Department of Nursing, Health Science College, Debre Markos University, Debre Markos, Amhara region, Ethiopia

**Keywords:** Birth preparedness, Complication, Readiness, Semi-pastoral, Pregnant women in southern Ethiopia

## Abstract

**Objective:**

The objective of this study was to determine the level of birth preparedness and complication readiness (BPCR) and associated factors among semi-pastoral pregnant women in southern, Ethiopia.

**Result:**

This dataset contains the full data collected from 746 pregnant women. Birth preparedness and complication readiness among women in southern Ethiopia was 27.1%. The main predictors for BRCP were attending formal education (AOR = 4.65, 95% CI 2.45–8.63), husband occupation [merchant (AOR = 3.83, 95% CI 1.52–9.64)], spouse attending formal education (AOR = 3.35, 95% CI (1.83–6.14), ANC visits > 4 times (AOR = 17.78, 95% CI 7.11–44.47). In addition, knowledge of women at least two danger signs during pregnancy, delivery and after delivery (AOR = 3.32, 95% CI 1.64–6.69), (AOR = 3.13, 95% CI 1.58–6.20) and (AOR = 3.75, 95% CI 1.93–7.28) respectively were significantly associated with BPCR. In conclusion, the proportion of BPCR among women in southern Ethiopia was found to be low.

**Electronic supplementary material:**

The online version of this article (10.1186/s13104-018-3539-7) contains supplementary material, which is available to authorized users.

## Introduction

Maternal mortality rate (MMR) remains a global public health concern especially in developing countries [1]. Worldwide, nearly 275,288 maternal deaths occurred due to pregnancy and related complications [2]. In 2000, millennium development goal (MDG) targeted for World Health Organization [1] member countries reducing MMR by 75% [3]. But still MMR was remains unacceptably high in developing countries particularly in sub-Sahara African countries [4, 5] which is 920 per 100,000 live births, and the lifetime risk of maternal death is 1 in 16 compared to 1 in 2400 in Europe [5]. Ethiopia is a country that experience high MMR rate [1]. It is the most sensitive indicator of the health disparities between poorer and richer nations, and for general growth. The effects of MM also have influences on other families like children [4, 6–9]. The survival status of all under-five children has significant association with child nutrition and other vital child health care practices seeking maternal involvement [10].

Low institutional deliveries, shortage of birth preparedness, the poor competence of health care providers, shortage of emergency obstetric services at facilities and week referral systems for obstetric emergencies were the common factors [11–14]. In Ethiopia, there is a minimal utilization of necessary maternal health services by pregnant women [12, 14–16]. This can be related to many factors identified by different studies are socio-demographic, cultural, communal, limited access to health facilities and poor quality of care in health facilities [12, 14–16]. The cause of maternal death was recognized as the three delays model [17] which is a delay in deciding to seek care (delay 1), delay in reaching the health facility (delay 2), and delay in receiving quality care once at the health facility (delay 3). And therefore, to reduce maternal death different strategies were initiated at the country wide. Of these, birth preparedness and complication readiness (BPCR) is one [12, 14–16]. In Ethiopia evidences show that the status of birth BPCR was low; Aleta Wondo district (17%) [18], Jimma Zone (23.3%) [19] and Basoliben District (26.9%) [20].

## Main text

### Method

#### Study area and period, study design and populations

A community-based cross-sectional study was conducted at Southern Ethiopia on August 2016. The setting is located 470 km away from the capital city of Ethiopia, Addis Ababa. According to the projected population estimate of 2016 the district has a total of 282,912 populations of these 42,707 women’s were expected to give birth [21]. All pregnant women who were permanently resided in the semi-pastoral of Bule Hora District were the study population.

#### Sample size and sampling procedure

Sample size was determined using single population proportion formula. We considered 17% level of compliance from the previous study [12]. We also considered a 5% level of significance (α) and 5% margin of error. Using these assumptions the sample size was calculated to be 339. After considering non-response rate (10%) and design effect (2), the final sample size obtained was 746. Participants were selected by multi-stage systematic random sampling from the population who were pregnant during the study period. The total number of pregnant women who lives in the study area was 3731 and we divided 3731 (sampling frame) by 746 (sample), so that a sampling fraction of 1/5 was obtained. To determine the order of the first respondent, we employed simple random sampling technique among the first five participants, and it was found to be the 3rd pregnant women. Thus, every 3rd participant starting from the first respondent was then included and interviewed until we get the required sample size.

#### Operational definition

##### Well prepared BPCR mother

If she reported at least three of five variables that she identified place of delivery, saved money, identify skilled provider at birth, identified a means of transport and identification of compatible blood donors in case of emergency.

##### Birth preparation

An arrangement, plan, or time preparing for delivery.

##### Complication readiness

It is a state of being prepared or willing for something that makes the situation difficult or the act of doing this (source JHPIEGO).

#### Data collection and quality management

Structured and pretested questionnaire were used for data collection. To maintain the data quality; pretest, training and supervision was done. Ten diploma nurse as data collectors and 2 BSc holder nurses as a supervisor were participated.

#### Data-analysis

The data were collected by interview on variables like, dependent variable: birth preparedness and complication readiness. Independent variables: maternal-factors (age, marital status, occupation, ethnicity, education, income, family size), reproductive factors (parity and history of still birth), paternal factors (occupation, education), health service factors (ANC use), obstetrics factors (gravidity) and knowledge factors (key danger sign, transportation, delivery cost and ANC) characteristics using structured and pretested questionnaire. The collected data were entered to Epidata version 3.1 and analyzed using SPSS version 22. Data were checked for consistency and completeness, and then descriptive and analytic computations were carried out. Bivariable (P value < 0.2 considered as a candidate variable multivariable binary logistic regression model was fitted to the data to identify variables associated with the dependent variable. Variables with P < 0.05 were considered statistically significant. SPSS version 20 was used to perform the analysis.

### Result

#### Socio-demographic characteristics of respondents

A total of 746 pregnant women were interviewed. Almost half, 384 (51.5%) of them were found between 25 and 34 years of age. Majority, 535 (71.7%) of the participants were protestant and, reflecting the ethnic composition of study setting which is Oromo (93.4%). Slightly less than half 347 (46.5%) of the pregnant women were illiterate, the majority of the respondents, 724 (97%), were married, 651 (87.3) of respondents parent were farmer and while 332 (44.5%) attended elementary education (Table 1).

**Table 1:** Socio-demographic characteristics of the respondents, among semi-pastoral pregnant women Bule Hora woreda, Oromia region, Ethiopia, August, 2016

Variables	Frequency	Percent (%)
Age		
16–24	196	26.3
25–34	384	51.5
35–49	166	22.3
Religious		
Protestant	535	71.7
Orthodox	89	11.9
Muslim	84	11.3
Others	38	5.3
Marital status		
Married	724	97
Other^a^	22	3
Ethnicity		
Oromo	697	93.4
Amhara	27	3.7
Others^b^	22	2.9
Occupation of women		
Housewives	550	73.7
Student	92	12.3
Farmer	27	3.6
Education level of woman		
Not attended formal education	332	44.5
Primary education (1–8)	91	12.2
Secondary education	219	29.4
College and above	10	1.3
Education level of husband		
Not attended formal education	258	38.2
Primary education (1–8)	249	33.3
Secondary education	187	25
College and above	25	3.4
Family size		
1–3	193	25.9
4–6	290	38.9
≥ 6	263	35.3
Parity		
0	22	2.9
1–2	255	34.2
>/3	469	62.9
History of stillbirth		
0	630	84.5
1	116	15.5
Monthly income (ETB)		
< 500	510	68.3
501–1000	130	17.4
Above 1000	106	14

^a^single, divorced, widowed

^b^protestant, catholic

#### Knowledge of danger signs

Regarding about knowledge on key danger signs during pregnancy showed that 198 (26.5%) and 158 (21.2%) of the study participants were aware of vaginal bleeding and severe fatigue respectively. During labor and delivery knowledge on severe vaginal bleeding, prolonged labor and retained placenta were 242 (32.4%), 337 (45.29%) and 298 (39.9%) respectively. Knowledge during post-natal period sever bleeding foul-smelling vaginal discharge and high fever was reported 265 (35.5%), 158 (21.2%) and 88 (11.8%) respectively. Of the total respondents, 304 (40.8%), 280 (37.5%) and 254 (34%) of them mentioned at least two key danger signs during pregnancy, delivery and after delivery respectively (Table 2).

**Table 2:** Knowledge of danger signs during pregnancy, labor and immediate postpartum period among semi pastoral pregnant women, Bule Hora woreda, Oromia region, Ethiopia, August 2016

Variables	Frequency			
Danger signs during pregnancy	Yes		No	
	No	%	No	%
Sever fatigue	158	21.2	588	78.8
Severe abdominal pain	142	19	604	81
Bleeding from the vagina	198	26.5	548	73.5
Fever	32	4.3	714	95.7
Unusual swelling of face/finger/legs	76	10.2	670	89.8
Sever and continued headache	151	20.2	595	79.8
Rapid or difficult breathing	61	8.2	685	91.8
Foul smelling vaginal discharge	68	9.1	678	90.9
Convulsions/fits	27	3.6	719	96.4
Loss of consciousness	58	7.8	688	92.2
Blurred vision	127	17	619	83
Danger signs in labor and delivery				
Heavy bleeding	242	32.4	504	67.6
Prolonged (> 12 h) labor	337	45.2	409	54.8
Vaginal tearing	75	10.7	671	89.9
Green or brown water coming from the vaginal	65	8.7	681	91.3
Water break and labor not induced within 6 h	67	9	679	91
Placenta not expelled within 1 h	298	39.9	448	60.1
Danger signs in postpartum				
Heavy bleeding	265	35.5	481	64.5
Bad smelling vaginal discharge	158	21.2	588	78.8
High fever	88	11.8	658	88.2
Painful urination	148	19.9	598	80.1
Hot swollen, painful breasts	186	24.9	560	75.1

#### Birth preparedness and complication readiness

Two hundred sixteen (29%) and 14 (1.9%) of the respondents had good knowledge on identifying place of delivery and blood donor respectively. In this study three and more steps from five where considered as a cutoff points to categorize whether pregnant women prepare for birth or not and that 202 (27.1%) of the study participants were found to be prepared for BPCR. One hundred seventy-two (23.1%) of the respondents were planned to identified a skilled birth attendant while 200 (26.8%) of the respondents were plan saving money in case of emergency. Similarly, 186 (24.9%) of the respondents arranged transport service for labor service (Additional file 1).

#### Role of husband during ANC and labour

Half, 366 (50.5%) and 404 (55.8) of the respondents Husband accompanied them to the Health facility visit. While, 108 (14.9%) and 102 (14.9%) of the husbands were cares children at homes and looked after the cattle at respectively (Additional file 2).

#### Factors associated with BPCR

Binary logistic regression showed that BPCR practice has a significant association with education status of pregnant women, educational and occupational status of their spouse, the frequency of the ANC is visiting as well as, knowledge of danger signs during pregnancy, labour and post-partum. The Multivariate logistic regression showed that BPCR was a significant association with variable like to attend formal education (AOR = 4.65, 95% CI 2.49–8.63), employment (AOR = 2.76 95% CI 1.10–5.54), merchant husbands (AOR = 3.83, 95% CI 1.52–9.64), the spouse with formal education (AOR = 3.35, 95% CI 1.83–6.14), ANC Visit more (Table 3).

**Table 3:** Bivariate and multivariate logistic regression of the likelihood to be prepared for birth and complications among semi pastoral pregnant women, Bule Hora, Oromia, Ethiopia, 2016

Variables	BPCR		OR (95% CI)	AOR (95% CI)	P value
	Yes	No			
Age of respondents					
16–24	49	147	1	1	
25–34	109	275	1.189 (1.08–1.7)	1.83 (1.55–3.22)	0.004
35–49	44	122	1.08 (0.064–1.736)	0.71 (.36–1.38)	
Education of woman					
Formal	157	166	7.95 (5.44–.11.59)	4.65 (2.49–8.63)	P < 0.001
Not formal	45	378	1	1	
Education of husband					
Formal	165	293	3.97 (2.65–5.94)	3.35 (1.83–6.14)	P = 0.002
Not formal	37	247	1	1	
Occupation of husband					
Farmer	151	500	1	1	
Merchant	27	19	4.65 (2.52–8.61)	3.83 (1.52–9.64)	P < 0.001
Employed	23	1	9.17 (3.25–25.89)	2.76 (1.10–5.54)	
Parity					
≤ 2	61	189	1	1	P = 0.14
> 3	132	344	1.28 (1.07–1.69)	1.39 (0.72–2.67)	
Knowledge at least two sign during pregnancy					
Yes	162	142	11.4 (7.72–17.02)	3.32 (1.64–6.69)	P = 0.001
No	40	402	1	1	
Knowledge at two danger signs during labor					
Yes	161	119	9.36 (6.37–13.76)	3.13 (1.58–6.20)	P < 0.001
No	41	425	1	1	
Knowledge of at least two danger signs after delivery					
Yes	153	101	13.69 (9.29–20.18)	3.75 (1.93–7.28)	P < 0.001
No	49	443	1	1	
ANC visit					
< 4	89	456	1	1	
≥ 4	113	14	19.9 (9.67–38.877)	17.78 (7.11–44.47)	P < 0.001

### Discussion

This study found that birth preparedness and complication readiness at western Oromia was accounted for 27.1% [22] of the pregnant women which is similar with studies done in Basoliben District, Goba district and Jima town of Ethiopia were 26.9, 29.9, and 23.3% respectively [15, 19, 23]. The finding is higher than findings at Aleta wondo 18.1% and Adigrat town, Tigray 21.2% [17, 24]. The discrepancy might have resulted from the difference between the residential areas in which the current incorporate mostly the rural kebeles than the previous one.

In other way, the current finding was lower than findings observed at Debrebirehan town and southwest Nigeria which was 53.9 and 51.6% respectively [25, 26]. This discrepancy might be due to the study area different. Debrebirehan is a zonal were most of the residents are educated compared with the current study area. In another case, the socio-economic status of Nigeria was better than Ethiopia.

This study also showed that women who had Knowledge about at least two danger signs during pregnancy, delivery and after delivering were 40.8, 37.5 and 34% respectively which is comparable to finding of Goba district danger signs during delivery and after delivery were 26 and 27% respectively but knowledge during pregnancy higher 66% [15]. The current finding has variation when compared with a study finding in Nepal knowledge on Obstetric danger signs during pregnancy, childbirth and postpartum were 34.8, 59.0 and 39.7% respectively [5, 19]. However it compared slightly higher to other studies done in Jimma town, the difference may be due to the fact that unlike the current study the former study considers woman is knowledgeable if only mention there and more danger signs which could decrease the number of respondents [19].

Another encouraging finding were identifying the place of delivery and saving money 29 and 26.8% respectively which are the most common practice of BPCR. Lower than the finding in Jima town 73.5 and 60.2% of the respondents planned to save money and arrange transport [19]. And a study in Uganda, Mbarara, and majority of the respondents identified the place of delivery and saved money [13]. However, planned to arrange blood donor and planned to be attended by skilled were 1.9 and 23.1% respectively, which was still low. These low levels of preparations were also reported in other prior studies in Ethiopia and other abroad countries. In the study conducted in Jima town west Ethiopia, 19.9% planned to arrange blood donor and 21.9% identify skilled attendant [19]. Similarly, in the study conducted in Sidama Zone, South Ethiopia in 2007, plan to arrange blood donor and identify skilled attendant was 2.3, 8.1% respectively [22]. Being BPCR is a new strategy which is yet to expand, low education level of women and the population as a whole may explain this low practice of the indication of PBCR among the study population.

Women education and Husband occupation were determinant factors for BPCR. Attending formal education among woman was 5 times more likelihood to increase BPCR than the counterpart (OR = 4.65 95% CI 2.49–8.63). Moreover, having spouse who is a merchant formally educated increased the likelihood of BPCR by 1.5 and 3.88 respectively which is comparable with previous findings in Ethiopia and abroad; where education, occupation, and income were among the factors affecting birth preparedness and complication readiness [15, 17, 22]. The possible reason for this could be due to the fact that being educated, being a merchant and employed increases access to information and are able to prepare for birth and complication readiness respectively.

Women who have more than three ANC visit were 17.78 times more likely to prepare for BPCR than the counterpart (P < 0.001, AOR = 17.78, 95% 95% CI 7.11–44.4) which was supported by a study conducted in Nepal [5]. Moreover, this study found that pregnant women knowledge towards at least two danger signs during, pregnancy (AOR = 3.32, CI 1.64–6.69), labor (AOR = 3.13 95% CI 1.58–6.20) and after delivery (AOR = 3.75, CI 1.93–7.28) were predictor for BPCR and this was supported by study findings done at Ethiopian country and abroad knowledge of danger signs was the strongest predictors of BPCR practice [3, 17, 22, 23]. This might be due to that knowledge of obstetric danger signs is essential to motivate women to seek skilled attendance at birth and prompt practices of another component of BPCR.

### Conclusions and recommendations

This study found that birth preparedness and complication readiness among pregnant women at southern Ethiopia was lower. Women education, husband-education, occupation, knowledge on danger-sign, and ANC visits were identified as factors affecting BPCR.

## Limitation

The limitation of this study was failing to consider temporal residence.

## Additional files

**Additional file 1.** Describe Birth preparedness and complication readiness status of pastoral women at southern Ethiopia 2016.

**Additional file 2.** Describe the role of Husband during ANC and Labour of their partners in southern Ethiopia 2016.

## Abbreviations

BPCR: birth preparedness and complication reediness
ANC: antenatal care
MMR: maternal mortality rate
MDGs: millennium development goal
WHO: World Health Organization
LB: live births
CI: confidence interval
EDHS: Ethiopian Demographic and Health Survey
