# Supplementary material for: Level and factors associated with birth preparedness and complication readiness among semi-pastoral pregnant women in southern Ethiopia, 2016
Source: BMC Res Notes. 2018 Jul 4;11:442. doi: 10.1186/s13104-018-3539-7 (PMC6030774; doi:10.1186/s13104-018-3539-7)
Supplement: Supplementary file 1 — Additional file 1. Describe Birth preparedness and complication readiness status of pastoral women at southern Ethiopia 2016. [file 13104_2018_3539_MOESM1_ESM.docx]

| **Variables** | | **Response** | **Frequency** | **Percentage** |
| --- | --- | --- | --- | --- |
| Types of Birth Plan | Identified a skilled birth attendant | Yes | 172 | 23.1% |
|  |  | No | 574 | 76.9% |
|  | Identified facility place of delivery | Yes | 216 | 29% |
|  |  | No | 530 | 71% |
|  | Arranged transport | Yes | 186 | 24.9% |
|  |  | No | 560 | 75.1% |
|  | Saved money in case of emergency | Yes | 200 | 26.8% |
|  |  | No | 546 | 73.2% |
|  | Identified blood donors | Yes | 14 | 1.9% |
|  |  | No | 732 | 98.1% |
