# Supplementary material for: Level and factors associated with birth preparedness and complication readiness among semi-pastoral pregnant women in southern Ethiopia, 2016
Source: BMC Res Notes. 2018 Jul 4;11:442. doi: 10.1186/s13104-018-3539-7 (PMC6030774; doi:10.1186/s13104-018-3539-7)
Supplement: Supplementary file 2 — Additional file 2. Describe the role of Husband during ANC and Labour of their partners in southern Ethiopia 2016. [file 13104_2018_3539_MOESM2_ESM.docx]

| **Variables** | **Response** | **Frequency** | **Percentage** |
| --- | --- | --- | --- |
| Role of husband during ANC | Accompanied her | 366 | 50.5% |
|  | Took care of domestic chores | 41 | 5.7% |
|  | Looked after the children at home | 108 | 14.9% |
|  | Looked after livestock | 102 | 14.1% |
|  | Others | 107 | 18.8% |
|  | Total (who have husband) | 724 | 100% |
| Role of husband during Delivery | Provide transportation or gave money | 43 | 5.9% |
|  | Accompanied her to health facility | 404 | 55.8% |
|  | Stayed at home with children | 18 | 2.8% |
|  | Bought baby clothes | 13 | 1.8% |
|  | Got some other person to take care of the home during the mother's absence | 176 | 24% |
|  | Looked after cattle or livestock | 19 | 2.6% |
|  | I don’t know | 51 | 7.4% |
